# Calculated parenteral initial treatment of bacterial infections: Infections in the ear, nose, throat and mouth and jaw area

**DOI:** 10.3205/id000058

**Published:** 2020-03-26

**Authors:** Bernhard Olzowy, Bilal Al-Nawas, Miriam Havel, Julia Karbach, Rainer Müller

**Affiliations:** 1 HNO-Zentrum Landsberg am Lech, Germany; 2 Klinik für Mund-, Kiefer- und Gesichtschirurgie, Universitätsmedizin Mainz, Germany; 3 Klinik und Poliklinik für HNO-Heilkunde, Klinikum der Universität München, Munich, Germany; 4 Klinik und Poliklinik für Hals-, Nasen- und Ohrenheilkunde, Universitätsklinikum Carl Gustav Carus Dresden, Germany

## Abstract

This is the sixth chapter of the guideline “Calculated initial parenteral treatment of bacterial infections in adults – update 2018” in the 2^nd^ updated version. The German guideline by the Paul-Ehrlich-Gesellschaft für Chemotherapie e.V. (PEG) has been translated to address an international audience.

The chapter deals with the antibacterial treatment of more severe infections of the ear, the nose, the throat and the maxillofacial region, including odontogenic and salivary gland infections.

## Zusammenfassung

Dies ist das sechste Kapitel der von der Paul-Ehrlich-Gesellschaft für Chemotherapie e.V. (PEG) herausgegebenen S2k Leitlinie „Kalkulierte parenterale Initialtherapie bakterieller Erkrankungen bei Erwachsenen – Update 2018“ in der 2. aktualisierten Fassung.

Es befasst sich mit der antibakteriellen Behandlung schwererer Infektionen im Hals-, Nasen-, Ohren- und Mund-Kiefer-Gesichtsbereich einschließlich odontogener und Speicheldrüseninfektionen.

## Introduction

Bacterial infections of the head and neck region often require the use of antibiotics. The decision for parenteral treatment depends on the severity of the infection, possible risk of spread (for example obstruction of the airways), comorbidities and individual application requirements. In some cases, even in severe infections, the good bioavailability of orally administered fluoroquinolones and clindamycin allows oral administration. This should be given preference as much as possible due to the greater ease of administering it. In contrast, initial treatment of mastoiditis, external otitis maligna, sinusitis with orbital or intracranial complications, epiglottitis, severe odontogenic abscess and cervical phlegmon should generally be with a parenteral antibiotic. As a rule, initial treatment must be calculated; however, in these serious diseases a diagnosis of the microbiological pathogen should be the aim. Sequential therapy is usually possible, i.e. oral treatment after clinical improvement.

The data on oral antibiotic treatment for minor infections of the head and neck region has improved in recent years. For appropriate recommendations, meta-analyzes of numerous randomized comparative studies with large patient populations are to hand. Such studies are rare for severe infections. Since severe infections are relatively uncommon and since there are ethical concerns regarding randomization of forms of treatment in which a statistically significant difference would be expected, an improvement of the data situation cannot to be expected in the medium term. Accordingly, current recommendations are essentially based on the likely pathogen spectrum, the local pathogen resistance situation and the action spectrum of the available antibiotics. Significant regional differences are to be expected with regard to the expected pathogen spectrum – depending on variables such as antibiotic use and vaccination situation. Current studies are scarce on the pathogen spectrum of severe bacterial infections of the head and neck region for the German-speaking countries. The current recommendations of the relevant specialist associations have been taken into account in the following recommendations [1], [2], [3], [4], [5].

## Otitis externa maligna and osteomyelitis of the base of the skull

Otitis externa maligna is a rare form of osteomyelitis that typically affects older diabetics and, more rarely, other immunosuppressed patients. It presents clinically as an otitis externa with unusually strong otalgia, otorrhea and granulation in the external auditory canal but cannot be cured with the usual local treatment of ear canal cleaning and antibacterial ear drops. In the process, cranial nerve failure can occur, especially pareses of the facial nerve. Fatal outcomes are recorded [6], [7], [8]. The suspected clinical condition should be clarified by means of CT, bone scintigraphy or MRI and in uncomplicated cases a microbiological diagnosis should be carried out after a 48-hour break of any antimicrobial treatment [9]. The disease is usually caused by *Pseudomonas aeruginosa* and only rarely by other pathogens [10], [11]. 

In hospitals, *Pseudomonas* isolates with a resistance to three or four of the common *Pseudomonas*-effective classes of antibiotics (3MRGN or 4MRGN) have a significant presence. However, colistin is almost always effective in vitro. In contrast, the resistance situation in *Pseudomonas* isolates from ear-smears in the outpatient care sector in Germany is relatively favorable. Sensitivity probabilities found during the 2013 PEG resistance study (specifically isolates from ear-smears) for outpatient or inpatient care were as follows: Levofloxacin 88% and 70%, ciprofloxacin 88% and 77%, meropenem 100% and 82%, piperacillin 98% and 84%, cefepime 98% and 90%, ceftazidime 98% and 87%, and colistin 100% and 100% respectively [12], [13]. 

A recently published meta-analysis of 30 case series concludes that initial combination treatment is superior to monotherapy. The authors recommend – if no relevant resistance is present - ceftazidime and ciprofloxacin for 3 weeks, followed by 3 weeks of ciprofloxacin p.o. as a treatment of choice [11]. In Germany, this combination should therefore be used at least in complicated cases (cranial nerve pareses) as the treatment of choice (recommendation grade B). Alternatively in uncomplicated cases, ceftazidime or piperacillin can be used in monotherapy (recommendation grade C). Initial parenteral monotherapy with a fluoroquinolone should only be considered due to the relatively unfavorable resistance situation for patients with an allergy to beta-lactams. Meropenem has no advantage over ceftazidime or piperacillin regarding *Pseudomonas* activity. Rather, the spectrum of action is unnecessarily broad. Aminoglycosides are unsuitable because of their limited bone penetration. Despite severe nephrotoxic reactions, colistin was re-approved in Germany in 2012 for parenteral treatment of multidrug-resistant Gram-negative pathogens The use in the context of calculated initial treatment should only be considered in complicated cases for patients with an allergy to beta-lactams, with the dosage adapted to the kidney function (recommendation grade C). Treatment duration should be about 6 weeks. More extensive bone necrosis may require surgical debridement of affected areas [14].

Two recent studies from China and the US report an increasing incidence of osteomyelitis of the lateral and central skull base with only minor pain symptoms and therapy-resistant otorrhea, especially in patients after ear operations but also those without any ear symptoms, in which, in addition to *Pseudomonas aeruginosa*, *Staphylococcus aureus*, also in particular MRSA was found as the causative agent [10], [15]. In these rare situations, the standard *Pseudomonas* antibiotics should be combined with a bone-active MRSA-effective antibiotic, for example linezolid or daptomycin (see chapter 10 [16]) (recommendation grade B).

## Mastoiditis

Acute mastoiditis is a relatively common complication of acute or chronic otitis media, in which there is a suppurative liquefaction of the trabeculae in the mastoid. It predominantly occurs in children, recently with an increasing incidence [17]. Only one study on mastoiditis in adults could be identified. The pathogen spectrum corresponds essentially to that of acute or chronic otitis media. In the following, the data on the expected pathogen spectrum in children is presented and extrapolated for the treatment recommendations for adults.

The serious clinical picture of acute mastoiditis should be differentiated from other fluid accumulations in the mastoid, which are also frequently described radiologically as mastoiditis. The diagnosis of acute mastoiditis is clinically based on redness, swelling and pressure pain over the mastoid and the resulting protrusion of the auricle [18]. It is mostly caused by pneumococci [17], [18], [19], [20], [21], [22], [23], [24], [25], [26]. According to studies in Finland and the USA, the introduction of vaccination against pneumococci has only resulted in a short-term lower incidence, followed by a marked increase in pneumococci with reduced susceptibility to antibiotics [20], [23]. Other frequently isolated pathogens are *Streptococcus pyogenes*, *Pseudomonas aeruginosa*, *Staphylococcus aureus* and *Haemophilus influenzae* [17], [19], [21], [22], [24], [26], [27] and more recently also *Fusobacterium necrophorum* [21], [28]. In children under two, *Streptococcus pneumoniae* dominates significantly. *Pseudomonas aeruginosa* and *Fusobacterium necrophorum* are almost exclusively isolated in children over the age of two [21], [22]. *Pseudomonas aeruginosa*, in particular, has to be expected in cases of recurrent otitis, tympanostomy tubes and otorrhea. The clinical progression also seems to be normal [26], [29], so that it seems justifiable in the absence of the mentioned criteria, in the context of an initial calculated antibiotic treatment to drop *Pseudomonas*-effective antibiotics in favor of antibiotics without *Pseudomonas* activity. Antibiotic treatment in this case should rely on amoxicillin/clavulanic acid, ampicillin/sulbactam, cefotaxime or ceftriaxone + clindamycin or levofloxacin. If *Pseudomonas aeruginosa* has also been detected, treatment with ceftazidime + clindamycin, piperacillin/tazobactam or meropenem should be considered. In the presence of a genuine allergy to beta-lactams, the combination ciprofloxacin + clindamycin is an option. A paracentesis accompanied by a microbiological pathogen diagnose, possibly with tympanostomy insert, should be performed. In case of complications, subperiosteal abscess or lack of improvement, an antrotomy or mastoidectomy should be performed [30], [31]. The duration of treatment is 7–10 days.

## Epiglottitis

While acute epiglottitis in the period prior to the introduction of the *Haemophilus influenzae* type B vaccine was primarily a pediatric disease caused by this pathogen, other pathogens are more commonly isolated today and adults are more frequently affected than children [32], [33], [34], [35], [36]. It is usually an acute, severe disease with rapid progression, which usually requires immediate inpatient admission, maybe even intensive care monitoring with the possibility of intubation or tracheostomy due to the risk of airway obstruction. In adults, the disease may be caused by pre-existing cysts, which is associated with more severe outcomes and an increased likelihood of requiring surgical intervention [37], [38]. Since any form of manipulation should be avoided in an emergency situation, pathogen diagnostics are rarely performed. Therefore, statements on the frequency of occurrence of certain pathogens are possible only with reservations. In adults, various types of streptococci dominate, while anaerobes and *Haemophilus influenzae* type B are isolated less frequently but in relevant frequency, quite often as part of a mixed infection [32], [33], [34], [35]. Individual case reports exist of acute epiglottitis caused by *Staphylococcus aureus* [39], *Haemophilus parainfluenzae* [40], *Corynebacterium diphtheriae* [41], *Mycobacterium tuberculosis* [42] and meningococci [43]. In addition, acute epiglottitis can also be caused by viruses or have a non-infectious cause. Since this is a life-threatening clinical scenario, the most frequent pathogens should be reliably covered by the calculated antibiotic treatment. Amoxicillin/clavulanic acid or ampicillin/sulbactam should be considered in the main [34], [35]. Alternative treatment options are ceftriaxone or cefotaxime + clindamycin or metronidazole [34], [35] and moxifloxacin. At the same time, high-dose corticosteroids should be administered initially [34], [35].

## Auricular perichondritis

Auricular perichondritis is characterized by the spread of acute inflammation of the outer ear to the perichondrium. Possible causes include scratch damage, mechanical trauma (othematoma, otostomy), surgical procedures with exposure of the ear cartilage, frostbite, burns, insect bites and piercing but in more than half of cases no cause can be identified [44]. By far the most common pathogen is *Pseudomonas aeruginosa*. According to one review, it occurs in 87% of infections following piercing [45]. The second most common pathogen is *Staphylococcus aureus*, followed by *Streptococcus pyogenes* and other streptococcal species. In individual cases, Enterobacteriaceae and enterococci have also been isolated [44], [45], [46], [47]. 

Material should be obtained for microbiological diagnostics before the start of treatment. Carrying out a Gram preparation, the result of which is often available within a few hours, can help prevent the use of antibiotics with an unnecessarily broad spectrum. In case of mild forms of Gram-positive cocci (*Staphylococcus aureus*, streptococci), ciprofloxacin p.o. should be given; clindamycin may be useful in cases of Gram-negative rods (usually *Pseudomonas aeruginosa*). More severe forms should be treated primarily parenterally because of the risk of abscess formation resulting in permanent deformation of the pinna. Piperacillin/tazobactam, cefepime + clindamycin and ceftazidime and ceftazidime (recommendation grade B) are the antibiotics of choice for treatment. Antiseptic treatment is also applicable. Ciprofloxacin and levofloxacin should only be used in cases of penicillin allergy due to the significantly higher proportion of fluoroquinolone-resistant strains (recommendation grade B). If there is no response to treatment, this may point to relapsing polychondritis or a resistant strain of *Pseudomonas* as the cause of the infection or cartilage necrosis requiring surgical debridement. The antibiotic susceptibility rates of *Pseudomonas aeruginosa* found in the 2013 PEG resistance study for outpatient (specifically ear-derived isolates) or inpatient care were as follows: Levofloxacin 88% and 70%, ciprofloxacin 88% and 77%, meropenem 100% and 82%, piperacillin 98% and 84%, cefepime 98% and 90%, ceftazidime 98% and 87%, and colistin 100% and 100% respectively [12], [13]. Once the antibiogram is available, treatment may possibly be continued orally. An incision is necessary if there is pus accumulation, in case of necroses gentle cartilage removal with best possible preservation of the auricle and possibly plastic reconstruction of the auricle after healing.

## Nasal boil

Nasal boil is a painful infection of the hair follicles in the nasal vestibule with phlegmonous spread to the tip of the nose, to the bridge of the nose, to the upper lip and along the bridge of the nose caused by *Staphylococcus aureus* in the sense of an endogenous infection from colonization of the nasal atrium [48], [49]. A study of the resistance status of the nasopharyngeal *Staphylococcus aureus* from nine European countries shows an MRSA frequency of 0–2.4% for adults. 5–15% of isolates were resistant to clindamycin [50]. In the 2013 PEG Resistance Study, all *Staphylococcus aureus* isolates from outpatient care contained 8% MRSA [51].

Outpatient treatment with topical antiseptics or antibiotics is usually sufficient in the case of the frequently occurring mild forms (folliculitis without liquefaction and without significant phlegmonous reaction in the surrounding tissue); if a tendency to spread develops, oral antibiotic treatment can be carried out in addition. If possible, the boil should be lanced. In cases of complicated progression, treatment is carried out intravenously and under inpatient care due to the risk of thrombosis of the angular vein with transmitted sepsis to the cavernous sinus [52]. If possible, antibiotic treatment is carried out with a penicillinase-resistant penicillin or a staphylococcal-effective narrow-spectrum cephalosporin. The antibiotic of choice is cefazolin. Flucloxacillin has a less favorable side-effect profile than cefazolin due to its hepatotoxic side effects [53]. Alternatively, cefuroxime, amoxicillin/clavulanic acid or ampicillin/sulbactam can be used but have an unnecessarily broad action spectrum. Clindamycin is used in patients with a penicillin allergy. If there is no improvement, the presence of MRSA should be investigated. A treatment duration of one week is usually sufficient. Moving to oral sequential therapy quickly is desirable. Suitable antibiotics are cefalexin, doxycycline and clindamycin.

## Peritonsillitis and peritonsillar abscess

In peritonitis, inflammation has spread in the connective tissue between a tonsil and the constrictor pharyngeal muscle. Peritonsillar abscess is understood to mean the generally one-sided liquefaction of phlegmonously inflamed peritonsillar tissue with accumulation of pus. Peritonsillar abscesses occur predominantly in young adults and more rarely in children. Clinically, it presents as protrusion of the tonsils accompanied by redness and swelling of the palatal arches. In addition, a uvula edema and jaw clamp may be present. 

A review published in 2013 with pooled data from 15 studies published between 1980 and 2012 provides a differentiated view of the probable spectrum of causative pathogen [54]. In the majority of cases, these are mixed infections. The most commonly isolated pathogens in monoinfections were group A streptococci (20–45%, but this may be a low frequency estimate due to prior antibiotic treatment) and fusobacteria (4–55%; the dominant pathogen in three Danish studies, especially among children). In mixed infections presumably especially Streptococci of the *Streptococcus anginosus* group (*Streptococcus intermedius*, *Streptococcus anginosus* and *Streptococcus constellatus*), which were isolated in up to 51% of cases, play an important role. In monoinfections *Staphylococcus aureus*, *Nocardia asteroids*, *Haemophilus influenzae*, *Arcanobacterium haemolyticum* and *Streptococcus pneumoniae* have been isolated occasionally or in isolated cases. In contrast, certain species often isolated in the context of mixed infections, especially Streptococci of the serological group C, *Peptostreptococcus* and *Prevotella* spp, are only of secondary importance for the disease process [54]. Subsequent publications, which address the pathogen spectrum, did not reveal many new aspects [55], [56], [57].

With regard to the selection of an adequate antibiotic treatment, two randomized comparative studies are available, both of which show no difference in the clinical course of the disease between the treatment arms. In one study, the efficacy of penicillin compared to penicillin + metronidazole [58] and in the other the efficacy of penicillin compared to ampicillin + sulbactam was tested [59]. However, the significance of both studies is limited because of the small number of cases (n=40 and 42 respectively) and simultaneous abscess drainage. In a non-randomized, prospective observational study with 117 patients after single needle aspiration, following monotherapy with penicillin new surgical intervention was required significantly more often than after monotherapy with amoxicillin/clavulanic acid or cefuroxime + metronidazole (14.7% vs. 4.7%) [60].

Treatment of peritonsillitis is by oral or parenteral antibiotic therapy. Peritonsillar abscesses are treated surgically and combined with peri- and postoperative antibiotic therapy. Whenever possible abscess aspiration, abscess incision and irrigation or abscess tonsillectomy are desirable. The recovered pus should be examined microbiologically [4].

Amoxicillin/clavulanic acid, ampicillin/sulbactam or cefuroxime + metronidazole should be used as first line antibiotics (recommendation grade A). All essential pathogens discussed in the literature as the cause of the disease are hereby covered. As alternatives in case of penicillin allergy, clindamycin or moxifloxacin may be considered (recommendation grade B), with the expectation of likely fusobacteria resistance in 16–25% and 7–53% of cases respectively [61]. A Canadian study reports that 32% of clindamycin-resistant strains were isolated from the streptococcal isolates from peritonsillar abscesses [57], whereas in Germany in 2013 only 3% of the isolates of *Streptococcus pyogenes* were clindamycin-resistant, according to the PEG resistance study and no resistance to moxifloxacin was found [12]. Cephalosporin monotherapy is not recommended as fusobacteria usually produce cephalosporinase [61]. A rapid change to oral administration of medication should be the target. The duration of treatment is around 5–7 days. In a prospective randomized study with 105 patients who were either treated with benzylpenicillin after abscess tonsillectomy or who discontinued any antibiotic treatment, no difference was found between subjective swallowing and pain scores and the progression of leukocyte count and C-score reactive protein [62] so that in uncomplicated cases after abscess tonsillectomy, immediate discontinuation of antibiotic treatment seems justifiable.

## Bacterial sinusitis and its complications

Only in severe cases or without spontaneous healing should acute rhinosinusitis be treated with antibiotics, which are then administered primarily orally (recommendation grade A) [63], [64]. In individual cases, indication for parenteral treatment may exist in patients with severe concomitant diseases, unusually severe progression or in the absence of improvement after oral antibiotic treatment. In such cases differential diagnostics should investigate the possibility of an invasive mycosis or non-infectious disease (such as granulomatous polyangiitis, early stage Wegener’s granulomatosis) and the indication for surgical intervention should be considered. With calculated parenteral antibiotic treatment, the most important causative agents of acute rhinosinusitis – *Streptococcus pneumoniae*, *Haemophilus influenzae*, *Moraxella catarrhalis*, *Streptococcus pyogenes*, *Staphylococcus aureus* and, in the case of a dentogenic cause, also various other streptococci and anaerobes [64] should be covered. In Germany, treatment with amoxicillin/clavulanic acid, ampicillin/sulbactam, cefotaxime or ceftriaxone + clindamycin; or moxifloxacin or, in the case of poorer anaerobic activity, levofloxacin is recommended. In patients with cystic fibrosis [65] and patients undergoing bone marrow transplantation [66], *Pseudomonas aeruginosa* must be considered a possible pathogen. In these cases, the use of piperacillin/tazobactam, ceftazidime + clindamycin or meropenem makes sense.

The various forms of chronic sinusitis are generally not treated with antibiotics but with topical steroids (recommendation grade A) and nasal irrigation. In the instance of primarily very pronounced cases or those that cannot be treated conservatively, surgical intervention should be considered. In the case of an odontogenic cause, the primary goal should be restoration of the dentogenic source. Concomitant oral antibiotic treatment may be useful in acute exacerbations (grade B recommendation) or with markedly purulent forms. In contrast, parenteral treatment should be reserved for patients with severe comorbidities or unusually severe progression. Since in chronic sinusitis there are numerous species of bacteria which may be the pathogen in question, treatment should be as targeted as possible. Commonly isolated pathogens include *Staphylococcus aureus*, streptococci, *Haemophilus influenzae*, various Enterobacteriaceae species (*Escherichia coli*, *Klebsiella pneumoniae*, *Klebsiella oxytoca*, *Serratia marcescens*, *Proteus mirabilis*), *Pseudomonas aeruginosa* and anaerobes [67], [68], [69], [70]. In calculated treatment, piperacillin/tazobactam, ceftazidime + clindamycin or levofloxacin or moxifloxacin can be used. 

The pathogen spectrum of odontogenic maxillary sinusitis is easier to calculate. Most are mixed infections caused by streptococci and anaerobes. *Moraxella* spp. and *Haemophilus* spp. are involved. Calculated treatment, which accompanies source control, is carried out either with amoxicillin/clavulanic acid, ampicillin/sulbactam or clindamycin [70]. In addition to antibiotic treatment – at the latest after disappearance of the acute symptoms – surgical restoration pf the odontogenic cause and possibly closure of a possibly present oral antrum connection is necessary.

With a corresponding risk history, multidrug-resistant pathogens must be expected in acute as well as in chronic sinusitis. In a retrospective study by Stanford University, paranasal sinus smears were analyzed over a period of just over than 20 years. The proportion of *Staphylococcus aureus* grown from the smears was 7.7%. The proportion of MRSA in the *Staphylococcus aureus* isolates increased from 1.7% (1990–1999) to 26.8% (2006–2010) [71], which is specific to the US and cannot be translated to Germany. Nevertheless, the use of vancomycin or linezolid in combination with meropenem, ceftazidime or ciprofloxacin should be considered if the risk and condition are severe. 

Orbital (60–75%), intracranial (15–20%) and bony complications (5–10%), which occur much more frequently as a result of acute rather than chronic rhinosinusitis, must be treated with parenteral antibiotics [64].

### Orbital complications of sinusitis

Orbital complications arise either through direct spread of inflammation through the lamina papyracea or through conduction in the veins. Deviating from the long-standing division according to Chandler, in which preseptal lid phlegmons, orbital cellulitis, subperiosteal abscesses, intraorbital abscesses and septic thrombosis of the cavernous sinus were understood as a stage-related progression [72], the preseptal lid phlegmon and septic thrombosis of the cavernous sinus are understood today as independent entities instead [64].

Pure eyelid phlegmons arise mostly not as a result of sinusitis but much more often in the context of infections of the upper respiratory tract, in dacryoadenitis or skin infections. Immediate assessment by an ophthalmologist is recommended. If proptosis, restriction of bulbus motility and visual disturbances (beginning with loss of red-green discrimination) have been excluded, in cases of low illness severity outpatient treatment with oral antibiotics may be used without resorting to imaging techniques. Severe postseptal infections are much rarer than preseptal eyelid phlegmons (about 80%) with a proportion of about 20% [73], [74]. In case of doubt or in the case of signs of an inflammation of the orbital contents, inpatient admission and parental antibiotic treatment should be initiated immediately and a contrast-enhanced CT or MRI should be performed [64]. 

The most common pathogens are *Staphylococcus aureus*, streptococci of the *Streptococcus anginosus* group and anaerobes. *Streptococcus pyogenes*, *Streptococcus pneumoniae*, *Haemophilus influenzae* and *Eikenella corrodens* are rarely but regularly isolated. *Pseudomonas aeruginosa*, *Escherichia coli*, *Klebsiella pneumoniae*, *Clostridium perfringens* and *Arcanobacterium haemolyticum* are also isolated [73], [74], [75], [76], [77], [78], [79], [80], [81], [82].

Antimicrobial treatment is initially high-dose with amoxicillin/clavulanic acid, ampicillin/sulbactam (recommendation grade A) or cefotaxime or ceftriaxone + clindamycin (recommendation grade B). In cases of penicillin allergy, combination of ciprofloxacin + clindamycin (recommendation grade B) is an option. Depending on the response to the treatment, changing quickly to oral administration of the medication may be possible [83]. Ciprofloxacin and clindamycin may also be administered orally in selected cases [84]. The duration of antibiotic treatment is about 3 weeks in total.

In general, there is also an indication for surgical intervention with restoration of the sinus source and possibly drainage of orbital abscesses. The obtained material should be microbiologically examined and the treatment continued in a targeted manner. In children younger than 2(–4) without abscess or with small medial abscesses and normal visual acuity who are responding rapidly to antibiotic treatment, conservative treatment alone may be sufficient [64].

### Intracranial complications of sinusitis

Epidural and subdural abscesses, meningitis, cerebritis and cerebral abscesses as well as septic thromboses of the superior sagittal sinus or cavernous sinus may occur in sinusitis through direct erosion of the skull base, conduction via diploic veins or by hematogenous scattering. The most common pathogens are streptococci (mainly streptococci of the *Streptococcus anginosus* group) and anaerobes, more rarely staphylococci [85], [86], [87], [88], [89]. Therapeutically, parenteral treatment with antibiotics, surgical restoration of the affected sinuses and – in the presence of abscesses – neurosurgical abscess drainage, each alongside microbiological pathogen diagnostics, is indicated [5], [64]. Cefotaxime or ceftriaxone, both in combination with metronidazole and linezolid, can be considered as first choice drugs in calculated treatment [5]. Those allergic to beta-lactams may be treated with levofloxacin as an alternative to cefotaxime/ceftriaxone. The duration of treatment is 30–60 days.

### Osseous complications of sinusitis, frontal osteomyelitis

Frontal osteomyelitis can occur after acute or chronic sinusitis, dental maxillary infections or after trauma. It occurs primarily in adolescents and is often associated with intracranial complications. The most common pathogens are *Staphylococcus aureus*, *Streptococcus pneumoniae* and beta-hemolytic streptococci (of the *Streptococcus anginosus* group in particular), *Haemophilus influenzae* and anaerobes. However, infections caused by *Pseudomonas aeruginosa*, *Serratia marcescens*, *Escherichia coli*, *Salmonella typhi*, *Pasteurella multocida* or fungi (*Aspergillus flavus* and Mucor) have also been described [90], [91].

Antimicrobial treatment should be initially high-dose with amoxicillin/clavulanic acid, ampicillin/sulbactam, cefotaxime or ceftriaxone (in combination with clindamycin) or with meropenem. In cases of penicillin allergy, moxifloxacin in combination with clindamycin is used (BEWARE: resistances in staphylococci). 

There is also the indication for surgical restoration of the affected frontal sinus and the removal of infested bone regions. Sinus puncture material, surgical drainage secretion and bloods are essential for microbiological examination. Upon receipt, targeted antibiotic treatment should be performed in accordance with the microbiological findings. Treatment takes place over a period of about 6 weeks.

## Odontogenic infections with a tendency to spread, possibly with local or systemic complications

Most odontogenic infections can be successfully treated with outpatient surgery without antibiotics. If the infection spreads or in case of patients with risk factors, calculated oral antibiotic treatment may be used [92]. It makes little sense to call for pathogen identification in dental practice in such uncomplicated odontogenic infections [93]. In severe odontogenic infections with a tendency to spread and the risk of local and systemic complications which must be treated parenterally, pathogen identification is indispensable, since in the event of a spread or complication treatment can then be targeted [94], [95], [96]. Mostly streptococci of the oral flora are found, more rarely staphylococci as typical aerobic pathogens, even in closed abscesses. Anaerobic or capnophilic pathogens such as *Prevotella* spp., Fusobacteria, Bacteroides species, Veillonella and Peptostreptococci are common [97], [98].

The data from most of the few existing studies show that pathogens with low resistance to penicillin and clindamycin are usually found in patients with odontogenic infections who have not been previously treated [94], [99], [100]. However, other authors report a 20% share of penicillin-resistant pathogens [101]. In complicated odontogenic infections in patients who have been previously treated and which require parenteral treatment, in 15–35% of cases penicillinase-producing pathogens were found [102], [103], [104] and in some cases critically high resistance rates to clindamycin of 25–45% [102], [103], [104], [105]. Prior treatment with antibiotics in particular seems to be a risk factor for the occurrence of penicillin-resistant pathogens [106], [107]. For severe odontogenic soft tissue infections that have already been pretreated with antibiotics, a higher proportion of isolates with resistance to penicillin and clindamycin must therefore be expected. From the above data it appears that almost all pathogens in the odontogenic region are sensitive to inhibitor-protected penicillins (such as amoxicillin/clavulanic acid). When assessing the resistance situation, however, one should bear in mind that the pathogenetic role of the identified bacteria has not been clarified. In life-threatening situations, carbapenems are the drug of choice for empirical treatment [101], [108], [109]. If allergy to beta-lactams is present, clindamycin should be used as an established alternative in monotherapy, with the above limitations. Moxifloxacin is another possible alternative [105], [110]. Note in this context the AWMF S3 guideline “Odontogenic infections” (registration number 007-006) [3].

## Osteomyelitis of the jaw

The most important forms with a bacterial cause are acute and secondary chronic osteomyelitis (odontogenic infection, pulpal and periodontal infection, infected extraction wounds) with leakage of pus, fistula and sequestration. Accordingly, there is a similar pathogen spectrum compared to odontogenic infections, with a high proportion of polymicrobial infections [111]. Actinomycetes are also often detected [112], [113]. Colonization or infection with multidrug-resistant Gram-positive pathogens has also been described in prolonged antibiotic pretreatment [114], [115]. Acute and secondary chronic osteomyelitis are treated both surgically and with antibiotics. This is to be distinguished from the rarer primary chronic osteomyelitis as a non-pustulant and chronic inflammation of unclear aetiology. In this form of osteomyelitis antibiotics, hyperbaric oxygen therapy, nonsteroidal anti-inflammatory drugs and glucocorticoids are used in addition to surgery [116]. 

Special forms of osteomyelitis, such as infected osteoradionecrosis or osteomyelitis induced by drugs such as bisphosphonates, corticosteroids and antineoplastic substances, are of particular importance because of their frequency and origin. Although the diseases are not primarily caused by bacteria, the bacterial superinfection almost always requires the most targeted adjuvant treatment possible. Because of the severity of the disease, initial antibiotic treatment is usually intravenous [117]. The pathogen spectrum is also similar to that of odontogenic infections [118].

The principle of osteomyelitis treatment consists of eradication of the source, removal of the infected and necrotic bone and empirical treatment, ideally with a pathogen-specific antibiotic. Because of the protracted progression, parenteral treatment is usually required. PMMA chains containing gentamicin have been used successfully for many years, especially in the chronic form [119]. Adjuvant antimicrobial treatment should consider the anaerobic pathogen spectrum in addition to the commonly isolated staphylococci [120]. Clindamycin or penicillin are recommended, however, after prior treatment pathogens with penicillin resistance are found more frequently [111]. Because of the potentially long and critical progression, pathogen diagnosis should always be sought. Some authors recommend that antibiotics should generally be continued orally for 4–6 weeks after surgery [116].

## Cervicofacial actinomycosis

The disease, which usually presents as a mixed infection with the pathogen *Actinomyces israeli*, can be treated well with antibiotics [121], [122]. Depending on the findings, additional surgery may be necessary. The microbiological findings or at least the histological confirmation of *Actinomyces* drusen is important. Actinomycetes are typically penicillin-sensitive. Penicillin is often given parenterally initially and then switched to oral therapy. In the case of a penicillin allergy, oral sequential therapy with doxycycline or parenteral administration of clindamycin or a cephalosporin is recommended. The importance of the accompanying obligate anaerobic flora is controversial [122], [123]. As with other chronic inflammations and because of poor penetration into the granulation tissue, treatment must be performed over a long time in high dosage. Data on the duration of treatment have not yet been collected for the cervicofacial form. A duration of up to 6 months is considered in complicated forms. For mild progressions or with sufficient surgical restoration, treatment duration of about 6 weeks is recommended [122].

## Sialadenitis

Sialadenitis is a bacterial or viral inflammation of the salivary glands. Sialadenitis often occurs as a superinfection after salivary gland dysfunction. Mostly the submandibular gland is affected. Secretion disorders of the salivary and mucous glands cause an increase in the viscosity of the saliva, which promotes the precipitation of inorganic substances. Saliva stones form, which can promote bacterial colonization and infection; they should be removed in the chronic phase [124]. Acute and chronic forms are distinguished. Sialadenitis in children is often caused by viruses (mostly mumps viruses, Parainfluenzae viruses, CMV), while in adults it is more likely to be caused by bacteria (staphylococci, streptococci and anaerobes). Recent publications have found evidence of an increased incidence of infection by Fusobacterium necrophorum (14%), especially in the presence of peritonsillar abscesses (91%) [125], [126]. Fusobacterium necrophorum can cause the serious clinical presentation of Lemierre syndrome. In the acute phase, in most cases, the focus is on conservative treatment. Because of the general symptoms which are often present, parenteral treatment or surgical relief is often required, which usually requires hospitalization. Serious bacterial infections must be treated parenterally with antibiotics. For mild infections, oral treatment is also possible. Studies from the period 1975–1985 described alpha-hemolytic streptococci and staphylococci as the main pathogens [127], [128], [129]. A recent case report highlights the importance of anaerobes in purulent sialadenitis [128], [129]. There are also recommendations for the use of cephalosporins, which, similar to fluoroquinolones, accumulate in the saliva [130] but their effectiveness against possible anaerobes is limited. The frequent penicillin resistance of sialadenitis pathogen leads to the recommendation to use aminopenicillin in combination with a beta-lactamase inhibitor or clindamycin [128].

## Summary of recommendations

Table 1 (Tab. 1) summarizes the treatment recommendations.

## Note

This is the sixth chapter of the guideline “Calculated initial parenteral treatment of bacterial infections in adults – update 2018” in the 2^nd^ updated version. The German guideline by the Paul-Ehrlich-Gesellschaft für Chemotherapie e.V. (PEG) has been translated to address an international audience.

## Competing interests

The authors declare that they have no competing interests.

## Figures and Tables

**Table 1:**
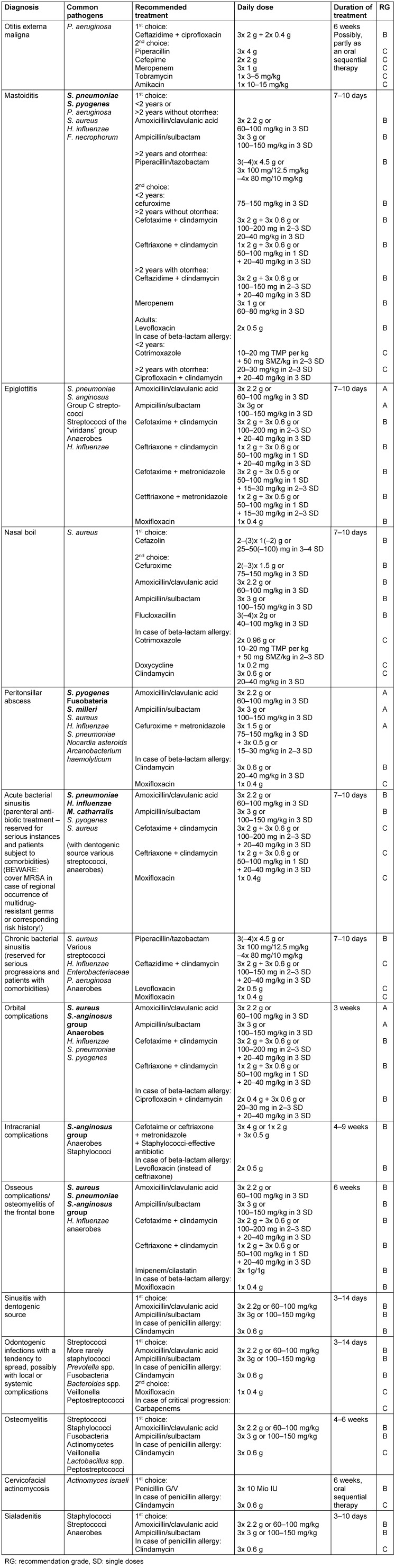
Treatment recommendations
